# Draft Genome Sequences of Two Alginate-Overproducing Variants of *Pseudomonas aeruginosa*, PAO1-VE2 and PAO1-VE13

**DOI:** 10.1128/genomeA.01031-13

**Published:** 2013-12-12

**Authors:** Yeshi Yin, T. Ryan Withers, Richard M. Niles, Shannon L. Johnson, Hongwei D. Yu

**Affiliations:** Department of Biochemistry and Microbiology, Joan C. Edwards School of Medicine at Marshall University, Huntington, West Virginia, USAa; Department of Pediatrics, Joan C. Edwards School of Medicine at Marshall University, Huntington, West Virginia, USAb; Progenesis Technologies, LLC, Huntington, West Virginia, USAc; Genome Science Group (B6), Los Alamos National Laboratory, Los Alamos, New Mexico, USAd

## Abstract

The small envelope protein MucE and the sensor kinase KinB are a positive and negative alginate regulator, respectively. Here, we announce the draft genome sequences of the alginate-overproducing variants *Pseudomonas aeruginosa* PAO1-VE2 (PAO1 with constitutive expression of *mucE*) and PAO1-VE13 (PAO1 with *kinB* inactivated). Both mutants were generated from a transposon mutagenesis screen.

## GENOME ANNOUNCEMENT

*Pseudomonas aeruginosa*, a Gram-negative bacterium, is the leading cause of morbidity and mortality in patients with cystic fibrosis (CF) (1). Alginate is an important virulence factor that promotes chronic lung infections in CF patients. Alginate regulation has been extensively studied in *P. aeruginosa* (1–4). One mechanism for the conversion to mucoidy or the overproduction of alginate is through activation of the intramembrane proteolysis leading to the degradation of wild-type MucA (3, 5, 6). The *mucE* and *kinB* genes encode a positive and negative regulator, respectively, of alginate overproduction in the wild-type *mucA* strains (5, 6). Here, we announce the draft genome sequences of two mucoid variants that are derivatives of the nonmucoid reference strain *P. aeruginosa* PAO1. Both strains were generated through a mutant screen with a mini-*Himar1* mariner transposon. Because of the transposon insertion, PAO1-VE2 has constitutive expression of a small envelope protein MucE (6), while PAO1-VE13 has an inactivated *kinB* gene (5).

In this study, cetyltrimethylammonium bromide (CTAB) and phenol-chloroform/isoamyl alcohol were used to extract the genomic DNA of PAO1-VE2 and PAO1-VE13. Paired-end sequencing libraries were generated according to the manufacturer’s protocol (Illumina, San Diego, CA). The genome sequencing was performed on an Illumina GAIIx. For PAO1-VE2, a total of 14,704,263 raw reads and 2,352,682,080 bp were obtained. For PAO1-VE13, a total of 13,964,486 raw reads and 2,234,317,760 bp were obtained. The sequence data were generated and assembled using the Illumina Pipeline version SCS 2.8.0 and paired using OLB 1.8.0. The sequences were then aligned and annotated using the reference genome of *P. aeruginosa* PAO1 (GenBank accession no. NC_2516.2) and the Novocraft NovoAlign version 2.07.13 software package. Further analysis of the genome sequence was performed using SAMtools version 0.1.15c for the generation of pileup after sorting and removing the duplicate reads. The analysis pipeline software was developed by CoFactor Genomics (St. Louis, MO, USA), and all specifics regarding the aligner algorithms can be obtained from the Novocraft website. The coverage of the generated sequences for PAO1-VE2 and PAO1-VE13 is 313.1× and 299.7× the reference genome. The number of base pairs saturated ≥8× for PAO1-VE2 and PAO1-VE13 are 6,263,842 (99.99%) and 6,263,748 (99.99%), respectively. The number of base pairs saturated <8× for PAO1-VE2 and PAO1-VE13 are 562 (0.01%) and 656 (0.01%), respectively. The two genomes were annotated and prepared for submission at the Los Alamos National Laboratory (LANL) using an automated annotation system built on the Ergatis workflow manager.

In comparison to the reference genome, we identified 71 heterozygous single nucleotide polymorphisms (SNPs) (0.3 ≤ count ratio ≤ 0.6) and 28 homozygous SNPs (count ratio > 0.6) for PAO1-VE2 and 70 heterozygous SNPs and 30 homozygous SNPs for PAO1-VE13. The count ratio is calculated using the following formula: (the number of times the reference base was observed)/(coverage at this base, counting all matches and mismatches). Among these SNPs, PAO1-VE2 and PAO1-VE13 share 66 heterozygous and 28 homozygous SNPs. While the majority of the heterozygous SNPs are located in the region corresponding to the probable bacteriophage PF1, two unique homozygous SNPs for PAO1-VE13 are located upstream of PA0149, encoding a probable sigma-70 factor, and PA4710, encoding a heme/hemoglobin uptake outer membrane receptor PhuR precursor.

### Nucleotide sequence accession numbers.

The draft genome sequences of the two mucoid *P. aeruginosa* strains have been deposited in GenBank with accession no. CP006831 (PAO1-VE2) and CP006832 (PAO1-VE13).
